# Hearing Sensitivity to Shifts of Rippled-Spectrum Sound Signals in Masking Noise

**DOI:** 10.1371/journal.pone.0140313

**Published:** 2015-10-13

**Authors:** Dmitry I. Nechaev, Olga N. Milekhina, Alexander Ya. Supin

**Affiliations:** Institute of Ecology and Evolution, Russian Academy of Sciences, Moscow, Russian Federation; University of Salamanca- Institute for Neuroscience of Castille and Leon and Medical School, SPAIN

## Abstract

The goal of the study was to enlarge knowledge of discrimination of complex sound signals by the auditory system in masking noise. For that, influence of masking noise on detection of shift of rippled spectrum was studied in normal listeners. The signal was a shift of ripple phase within a 0.5-oct wide rippled spectrum centered at 2 kHz. The ripples were frequency-proportional (throughout the band, ripple spacing was a constant proportion of the ripple center frequency). Simultaneous masker was a 0.5-oct noise below-, on-, or above the signal band. Both the low-frequency (center frequency 1 kHz) and on-frequency (the same center frequency as for the signal) maskers increased the thresholds for detecting ripple phase shift. However, the threshold dependence on the masker level was different for these two maskers. For the on-frequency masker, the masking effect primarily depended on the masker/signal ratio: the threshold steeply increased at a ratio of 5 dB, and no shift was detectable at a ratio of 10 dB. For the low-frequency masker, the masking effect primarily depended on the masker level: the threshold increased at a masker level of 80 dB SPL, and no shift was detectable at a masker level of 90 dB (for a signal level of 50 dB) or 100 dB (for a signal level of 80 dB). The high-frequency masker had little effect. The data were successfully simulated using an excitation-pattern model. In this model, the effect of the on-frequency masker appeared to be primarily due to a decrease of ripple depth. The effect of the low-frequency masker appeared due to widening of the auditory filters at high sound levels.

## Introduction

Many real-world sounds have time-varying spectra. Spectrum variations in time may be important cues for discrimination spectro-temporal patterns of acoustic stimuli. However, real-world sounds typically occur simultaneously with other sounds, which might be considered as interfering background noises. Therefore, it is important to understand how variations in the frequency spectra are detected by the auditory system in background noise.

The detection of variations in frequency spectra has been investigated in detail for pure tones, both in quiet and under conditions of background noise. In quiet, detection of as small variations of tone frequency as 0.2% has been reported [1]. Subsequent data showed similarly low thresholds for the difference limens for frequency (DLF) and difference limens for change (DLC) tasks at optimal carrier frequencies (around 2 kHz), and somewhat higher but still low for frequency modulation difference limens (FMDL): 0.4–0.5% [2–5].

Masking noise impairs pure-tone frequency discrimination. It was shown [6] that frequency discrimination worsened with decreasing signal/noise ratio. Experiments using notched noise have shown that both on-frequency noise and noise in remote frequency regions impair frequency discrimination [7], with noise centered below the signal tone frequency being more effective than noise centered above the signal tone frequency [8]. However, noise centered above the signal tone frequency impaired frequency discrimination too [4]. Poorer frequency discrimination was observed in simultaneous low-pass noise than in white noise [9]. Maskers centered far above the signal tone frequency produced no effect on frequency discrimination [10].

Considering that many natural sounds have wider and more complicated frequency spectra than pure tones [11], thresholds for ripple phase shift in spectra with frequency-proportional ripples were measured; the rippled-spectrum signals were considered as representative of multi-component signals. In that study, a test signal with ripples shifting up and down along the frequency scale should be discriminated from a comparison signal with constant ripple positions. The ripple shift thresholds were more than 1% at an optimal ripple density and up to 2–3% at both lower and higher ripple densities; i.e., nearly an order of magnitude higher than DLFs and several times higher than FMLD for pure tones. The difference between shift thresholds for pure tones and rippled spectra were explained using an excitation-pattern (internal spectrum) model. The pitch for pure tones and the absence of pitch for spectra with non-constant (frequency-proportional) ripple spacing may also play a role in different shift thresholds.

The influence of masking noise on rippled spectrum resolution has previously been described [12–14] but resolution was investigated by measuring the highest resolvable ripple *density*, but not minimal resolvable ripple *shift*. These are different capabilities, because even at a resolvable ripple density, the ripple shift may be non-resolvable.

The aim of the present study was to measure the ability of the auditory system to detect shifts in rippled spectrum along the frequency axis under conditions of simultaneous masking noise. We supposed that this study might provide data on influence of noise on discrimination of complex-spectrum signals and possible mechanisms of this influence. Rippled-spectrum signals may be considered as representative of complex-spectrum signal and can be quantitatively characterized based on a limited number of parameters: the frequency range, ripple density, phase, and depth. We used ripple spectra with frequency-proportional (not equally spaced) ripples, assuming that this pattern better corresponds to the variation of auditory filter bandwidth with frequency, which is nearly frequency-proportional within a major part of the frequency range of hearing [15, 16].

## Methods

### Listeners

Eight listeners (laboratory staff and volunteers) participated in this study. The listeners were 23–63 yr old individuals with hearing thresholds of less than 20 dB over the range 1–4 kHz. All listeners had long previous experience in psychoacoustic experiments with complex-spectra discrimination.

The experiments were approved by Ethic Committee of the Institute where the study was carried out, for sounds of SPLs of not more than 100 dB and everyday sound exposure levels (SELs) of not more than 120 dB as compliant with Sanitary Normative SN2.2.4/2.1.8.562–96.

### Signals

**Fig 1:**
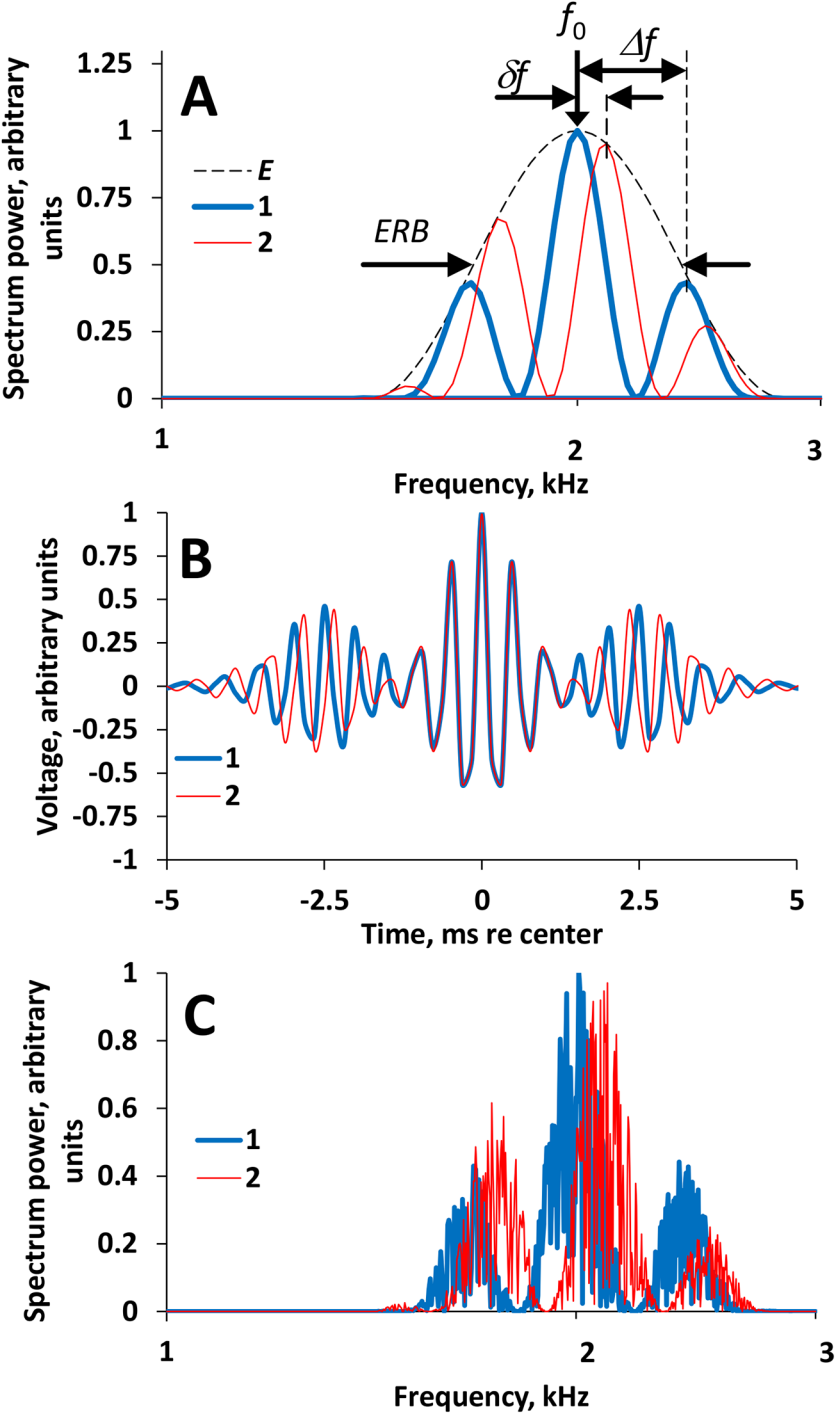
Test signal spectra. **(A**) Rippled filter shapes. 1 and 2—filters with ripples shifted relative one another, *E*—common envelope for the both filter shapes. *f*_0_—center frequency (2 kHz), *ERB*—equivalent rectangular bandwidth (0.5 oct), *Δf*—ripple spacing (20% of the center frequency), *δf*—ripple-phase shift (in the illustrated case, 5% of the center frequency). On the logarithmic frequency scale, frequency-proportional ripples look equally spaced. (**B**) temporal transfer functions (the finite impulse responses) of the filter shapes presented in (A), 1 and 2, respectively. Apart from the central segment (approximately from –1.5 to 1.5 ms), side segments (approximately from –5 to –1.5 and from 1.5 to 5 ms) are characteristic of the transfer function of rippled filter shapes. (**C**) Examples of spectra of 400-ms long samples obtained with the filters 1 and 2, respectively; spectra 1 and 2 satisfactorily reproduced the filter shapes 1 and 2 in (A).

The test signals were band-limited rippled noises with frequency spectra as shown in Fig 1. The envelope of the power spectrum of the signal was a one-octave wide cycle of a cosine function on a logarithmic frequency scale, centered at 2 kHz (*f*_0_ in Fig 1A). This center frequency resulted in the best sensitivity to ripple shift [11]. The equivalent rectangular bandwidth (ERB) of this band was 0.5 octaves (*ERB* in Fig 1A). The cosine envelope was used to avoid effects of sharp spectrum edges, which might influence the resolution of the ripple patterns [17].

The ripples within the cosine envelope were defined by a cosine function, as shown in Fig 1A. The ripple spacing (*Δf* in Fig 1A) was a constant proportion of the ripple center frequency (the ripples were equally spaced on a logarithmic scale). A convenient metric for this type of ripple pattern is the number of ripples per octave (rpo), hereafter referred to as the ripple density *D*. In the present study, *D* was 3.5 rpo. According to [11], this ripple density provides the best (lowest) thresholds for ripple shifts. At this ripple density, the ripple relative spacing *Δf*, defined as a ratio of the ripple absolute spacing to the center frequency of the ripple, was 0.2 (dimensionless units).

Thresholds for detecting ripple shifts along the frequency scale (*δf* in Fig 1) were measured. Only the ripples were shifted, leaving the spectrum envelope unchanged. In the measurement procedure, two signals were used: a test and a comparison signal (see below, experimental procedure). The test signal was a rippled-spectrum noise burst lasting 2.4 s. During this burst, every 0.4 s, the ripples shifted up and by *δf* and then back down by the same *δf*, i.e., one of the rippled spectra presented in Fig 1A was replaced with another spectrum with a shifted position of ripples on the frequency scale, and back. Thus, each test signal contained six ripple shifts (three up/down cycles of shifts). During the shifts, the spectrum did not exceed the signal passband. The rather long presentation of each of the noise versions (0.4 s) was necessary because at shorter durations, the difference between the rippled-spectrum versions could not be successfully extracted from fluctuations intrinsic in the noise [18]. Signal levels were 50 or 80 dB SPL.

The comparison signal was a rippled-noise burst lasting 2.4 s with the same spectrum and the same level as the test signal however without ripple shifts.

The signals were digitally generated at a sampling rate of 32 kHz. Details of the generation routine were described earlier [11]. The signal generation routine was as follows. The necessary digital filter shapes were generated for the test signal (Fig 1A). The two filters had different ripple phases, but were the same in all other respects (1 and 2 in Fig 1A). Inverse Fourier transforms of these filter shapes produced the temporal transfer functions (the finite impulse responses) of the filters (Fig 1B). A wide-band signal was generated as a random digital sequence uniformly distributed within a range of ±1; this sequence has a flat frequency spectrum up to *r*/2, where *r*–sampling rate. This wide-band signal was convolved with one of the temporal transfer functions of the filters. The convolution produced a filtered signal (Fig 1C). Every 0.4 s the wide-band signal (random digital sequence) was redirected from one of the filter inputs to another. The final signal waveform was obtained by summing the filter outputs. This operation resulted in a ripple shift every 0.4 s (spectra 1 and 2 in Fig 1C).

The spectra of the signal of a limited duration (0.4 s presentation of each rippled-spectrum version) did not precisely reproduce the filter forms because of the intrinsic fluctuations in the noise. However, the signals satisfactorily reproduced the spectrum envelope and rippled pattern (Fig 1C).

**Fig 2:**
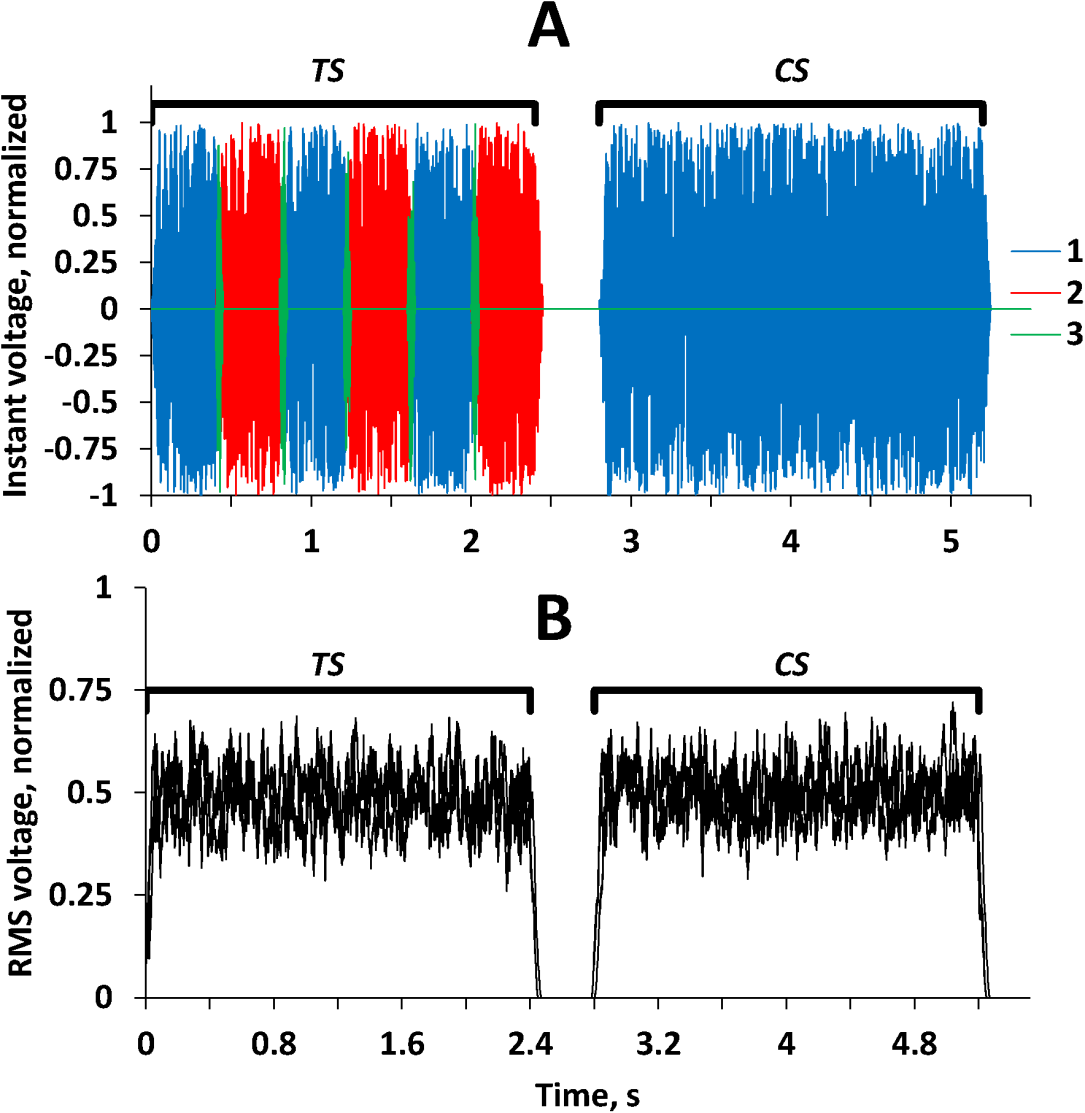
Succession of the test and comparison signals in time domain. **(A**) An arbitrarily chosen example of test signal (*TS*) and comparison signal (*CS*). 1 and 2—signals with ripple phases shifted relative one another, 3—transition segments. In the test signal, spectra 1 and 2 replaced one another every 400 ms, in the comparison signal, only filter 1 was used. (**B**) RMS of the signals averaged by a 20-ms sliding window; three arbitrarily chosen examples of test signal (*TS*) and comparison signal (*CS*) overlapped. Note the absence of RMS fluctuations associated with the spectrum-to-spectrum transitions (at 0.4, 0.8, etc. s) in the test stimulus and no difference of RMS patterns of the test and comparison signals.

Importantly, the phase-reversal was done by switching the inputs, not the outputs, of the filters; therefore, transitions from one spectrum to the other were determined by the filter transfer functions and the signal spectrum never fell outside the filter passband, i.e., no wide-band transients occurred at phase shifts. Also, at the ripple-phase shifts, the signal root-mean-square (RMS) had no fluctuations exceeding background fluctuations intrinsic in noise (*TS* segments in Fig 2A and 2B).

The comparison signal was generated using the same routine as for the test signal. However, instead of two filters with different ripple phases, one of the filters was used. In this case, the generated signal contained no ripple shift throughout all its duration of 2.4 s (*CS* time window in Fig 2A and 2B).

### Maskers

**Fig 3:**
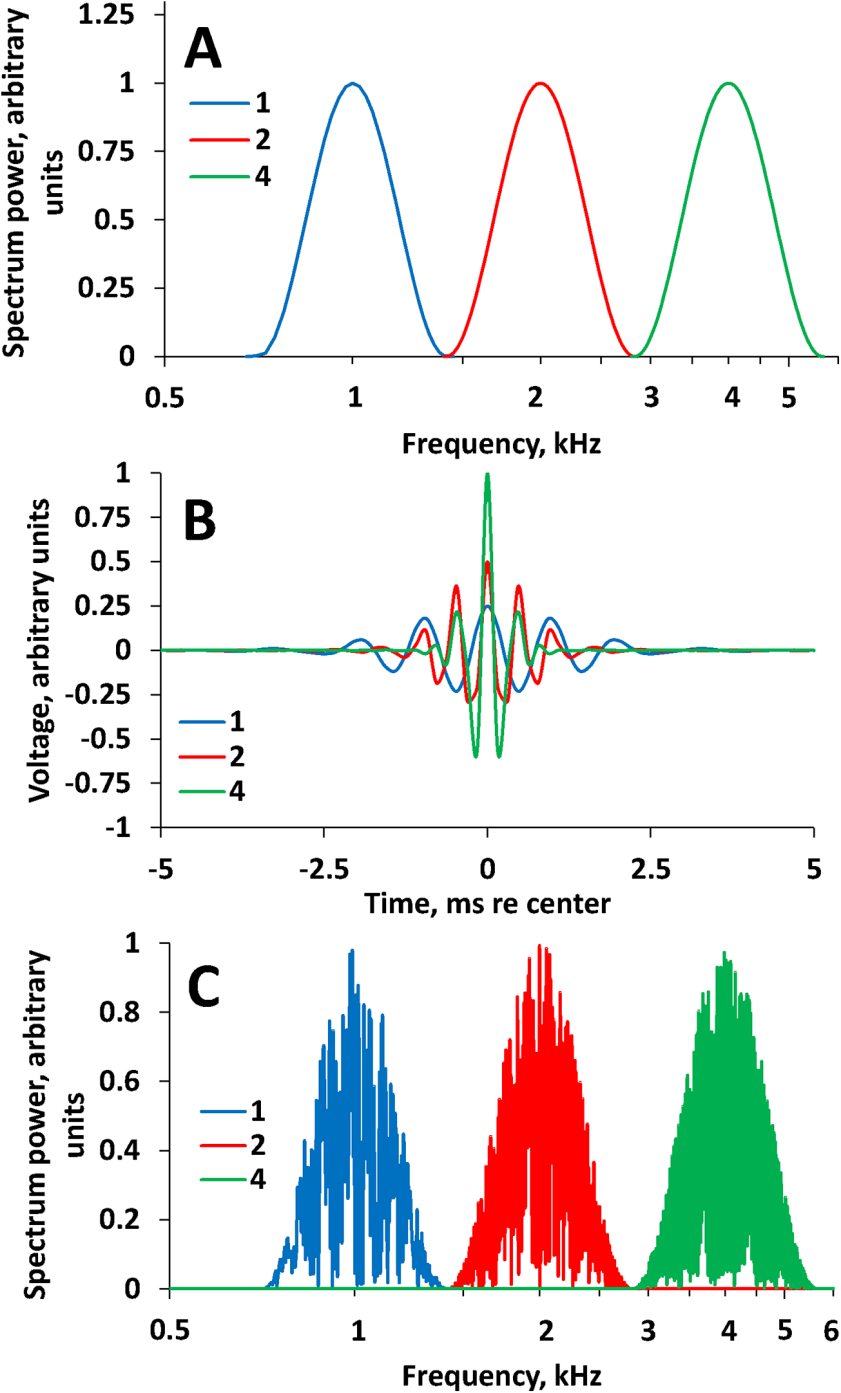
Masker spectra. **(A**) Filter shapes for 1, 2, and 4-kHz centered maskers, as indicated in the legend. (**B**) temporal transfer functions (the finite impulse responses) of the filter shapes presented in (A), for 1, 2, and 4-kHz centered filters, as indicated in the legend. The transfer functions do not feature side segments characteristic of rippled filters (see Fig 1). (**C**) Examples of spectra of 400-ms long samples obtained with the filters centered at 1, 2, and 4 kHz, as indicated in the legend. Although maskers were played during 2.4 s, 400-ms samples were taken to exemplify spectra for better comparison with Fig 1.

The masker spectra were centered at frequencies of 1, 2, or 4 kHz, i.e., 1 octave below, on, or 1 octave above the center frequency of the signal, respectively (Fig 3). Below, these maskers are referred to as low-, on-, and high-frequency maskers, respectively. All maskers were band-limited noises with frequency spectra corresponding to a one-octave cycle of a cosine function on a logarithmic frequency scale; e.g., for the on-masker, its spectrum was the same as the envelope of the signal. The masker duration was 2.4 s during the test signal and 2.4 s during the comparison signal (simultaneous maskers). Masker level varied from 30 to 100 dB SPL. Thus, at the maximum masker level (100 dB SPL), the sound exposure level (SEL) was 100.7 dB each trial and 115 to 117 dB (depending on the number of trials) each session.

The maskers were generated using the same routine as for signal generation, i.e., by convolution of a wide-band signal (random digital sequence) with the temporal transfer function of one of the filters (Fig 3B). Unlike test signal generation, only one filter was used to generate a 2.4-s long masker.

### Experimental procedure

A two-alternative forced-choice procedure with feedback was used. In each trial, the test and comparison signals were presented, each lasting 2.4 s, with a 0.4-s interval between them (see Fig 2). The order of signal presentation (first test, second comparison, or vice versa) varied randomly trial-by-trial (Fig 2 exemplifies the first test, second comparison case). In control runs, only the test and comparison signals were presented in random order. In masking runs, masker noise of 2.4 s in length was played simultaneously with both the test and comparison signals. The listener was asked to detect any periodic modifications in the noise occurring every 0.4 s and report which signal in the pair, the first or the second, was the rest, i.e., featured the modifications.

The ripple shift in the test signal was varied using an adaptive one-up, three-down procedure. After three successive correct responses, the phase shift decreased by 0.5% of the center frequency; after each mistake, the phase shift increased by 0.5% of the center frequency. This procedure brings the value of phase shift needed to achieve 79% correct. Maximal available ripple shift was 10% of the center frequency because at the ripple spacing of 20%, shifts more than 10% were equal to a lesser shift in the opposite direction.

Every run was initiated with a ripple shift well above the anticipated threshold. A part of the run until the first mistake was considered to be a warming-up segment. A part of the run that began from the next reversal (after three corrected responses) was considered as a measurement segment. The procedure lasted until eight reversals occurred. The ripple shift values at the eight reversals were averaged. The result was adopted as an estimate of the ripple shift threshold for this particular run.

For each combination of signal and masker parameters, the measurements were repeated three times for each of eight listeners. The results of the 24 measurements for every combination of the signal and masker parameters were averaged to yield a final threshold estimate as a mean with a standard error (SE).

### Instrumentation

All the signals were digitally synthesized on a standard personal computer using a custom-written program (virtual instrument) designed using LabVIEW software (National Instruments, Austin, TX, USA).

The digitally generated signals were digital-to-analog (D/A) converted using a 16-bit D/A converter in a data acquisition board NI-USB-6251 (National Instruments, Austin, TX). The analog signals were power-amplified, attenuated, and diotically played through HD580 headphones (Sennheiser, Wedemark, Germany). The frequency response of the headphones varied not more than 1.5 dB within each 0.5-octave band of the signal, measured using a Testo 816 noise-level meter (Testo AG, Lenzkirch, Germany) equipped with a 0.5-in. microphone terminated through a 6-cm^3^ coupler. The sound level of the signals was measured in the same manner.

## Results

### Baseline ripple shift threshold

Baseline shift thresholds were measured for signal levels of 50 and 80 dB SPL. The final threshold estimates (mean and SE) were 1.05 ± 0.06% of the center frequency for the 50 dB and 1.13 ± 0.06% of the center frequency for the 80 dB level. The difference between these two thresholds of 0.08 ± 0.08(SE) % did not reach the standard of statistical significance (*p* = 0.15).

### Effects of on-frequency maskers

**Fig 4:**
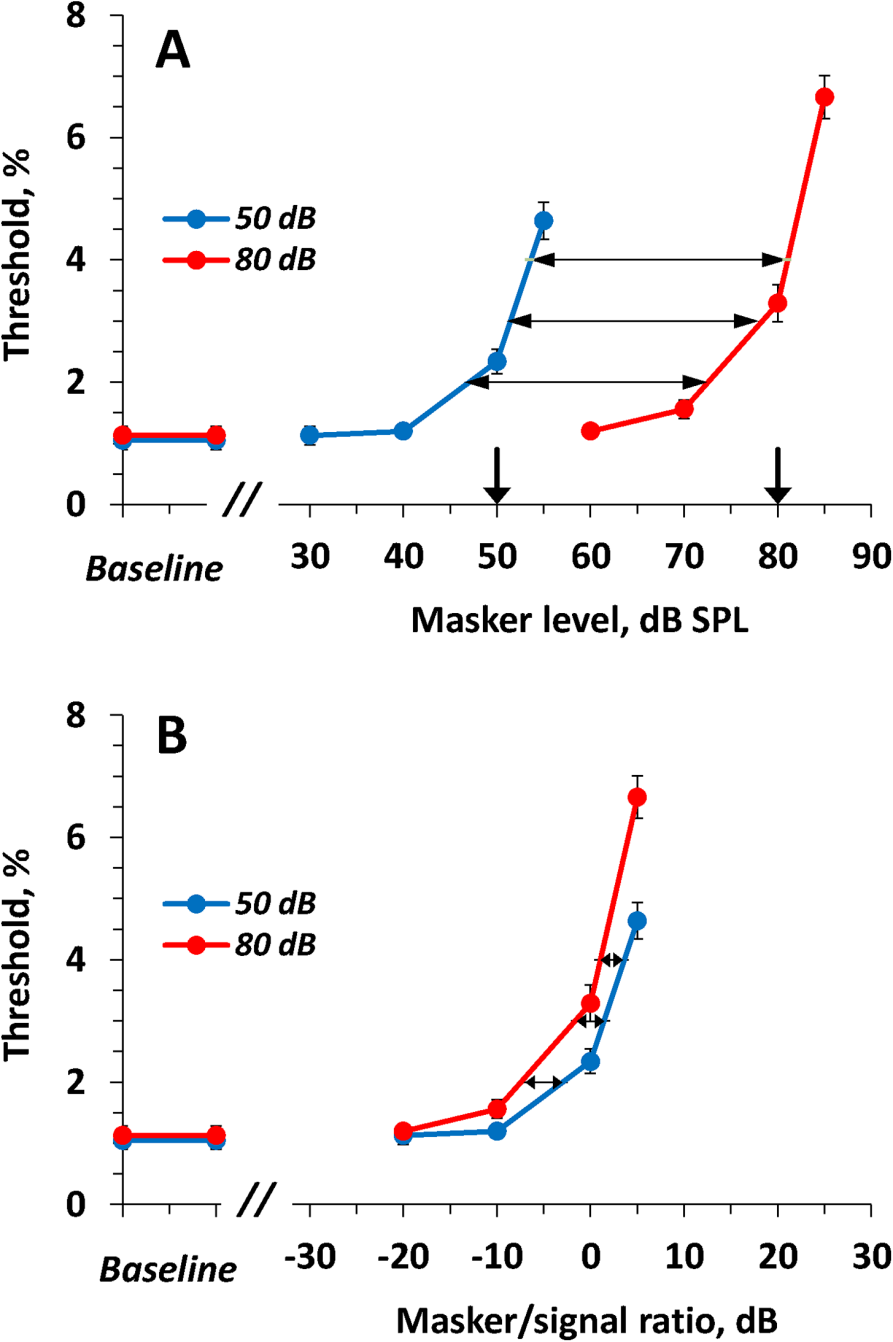
Dependence of ripple shift threshold on the on-frequency masker level. **(A)** Thresholds as a function of masker SPL. Functions for the 50 and 80 dB SPL test levels are as indicated in the legend. Error bars—SE. Downward pointing arrows depict the levels of the signals (50 and 80 dB). Horizontal double-headed arrows indicate the shift of the functions relative to one another at threshold values of 2, 3, and 4%. **(B)** The same data presented as functions of masker/signal ratio. Variation of masker/signal ratio keeping masker SPL constant (by change of signal SPL by 30 dB) resulted in gross change of thresholds (A) whereas variation of masker SPL keeping masker/signal ratio constant (by joint variation of masker and signal SPLs) resulted in minor change of thresholds (B).

The effects of on-frequency maskers were examined for signal levels of 50 and 80 dB SPL. The masker levels were varied from 30 to 90 dB SPL. The results are presented in Fig 4. At certain masker levels, the masker resulted in an increase of the ripple shift threshold. This effect depended on both the test and masker levels.

For the signal level of 50 dB SPL, a statistically significant difference (mean ± SE = 1.29 ± 0.11%, *p* < 0.001) from the baseline threshold was observed at a masker level of 50 dB SPL (Fig 4A), i.e., at a masker/signal ratio of 0 dB (Fig 4B). The threshold steeply increased with further increasing masker level. No sift was detectable up to the available maximum of 10% at masker levels of 60 dB SPL (masker/signal ratio of 10 dB) and higher. Note, Fig 4B presents thresholds as a function of masker/signal ratio, not signal/masker ratio. Otherwise, plots in Fig 4A and 4B looked inversed relative one another.

For the signal level of 80 dB SPL, a statistically significant threshold increase (mean ± SE = 0.43 ± 0.13%, *p* < 0.001) was observed at a masker level of 70 dB SPL (masker/signal ratio of –10 dB). No shift was detectable up to the available maximum of 10% at masker levels of 90 dB SPL (masker/signal ratio of 10 dB) and higher.

To compare the two threshold-vs.-masker level functions, we measured the distance between the thresholds along the masker level scale at several threshold values: 2, 3, and 4%, as shown in Fig 4A. The masker levels corresponding to these threshold values were obtained through interpolation between the nearest experimental points. The shifts at the threshold values of 2, 3, and 4% were 26, 27, and 27.5 dB, respectively with a mean of 26.8 dB.

The same data plotted as threshold versus masker/signal ratio (Fig 4B) showed substantial similarity between the two functions. Within a masker/signal ratio range from –10 to 0 dB, threshold increased by 1.14 ± 0.12 (SE) % (*p* < 0.001) for the 50-dB signal and by 1.73 ± 0.12 (SE) % (*p* < 0.001) for the 80-dB signal Threshold increased more with increasing masker/signal ratio for ratios from 0 to 5 dB, and no shift was detectable at ratios of 10 dB and higher. The shifts of the two functions relative to one another at threshold levels of 2, 3, and 4% were 4, 3, and 2.5 dB, respectively, with a mean of 3.2 dB.

### Effects of low-frequency maskers

**Fig 5:**
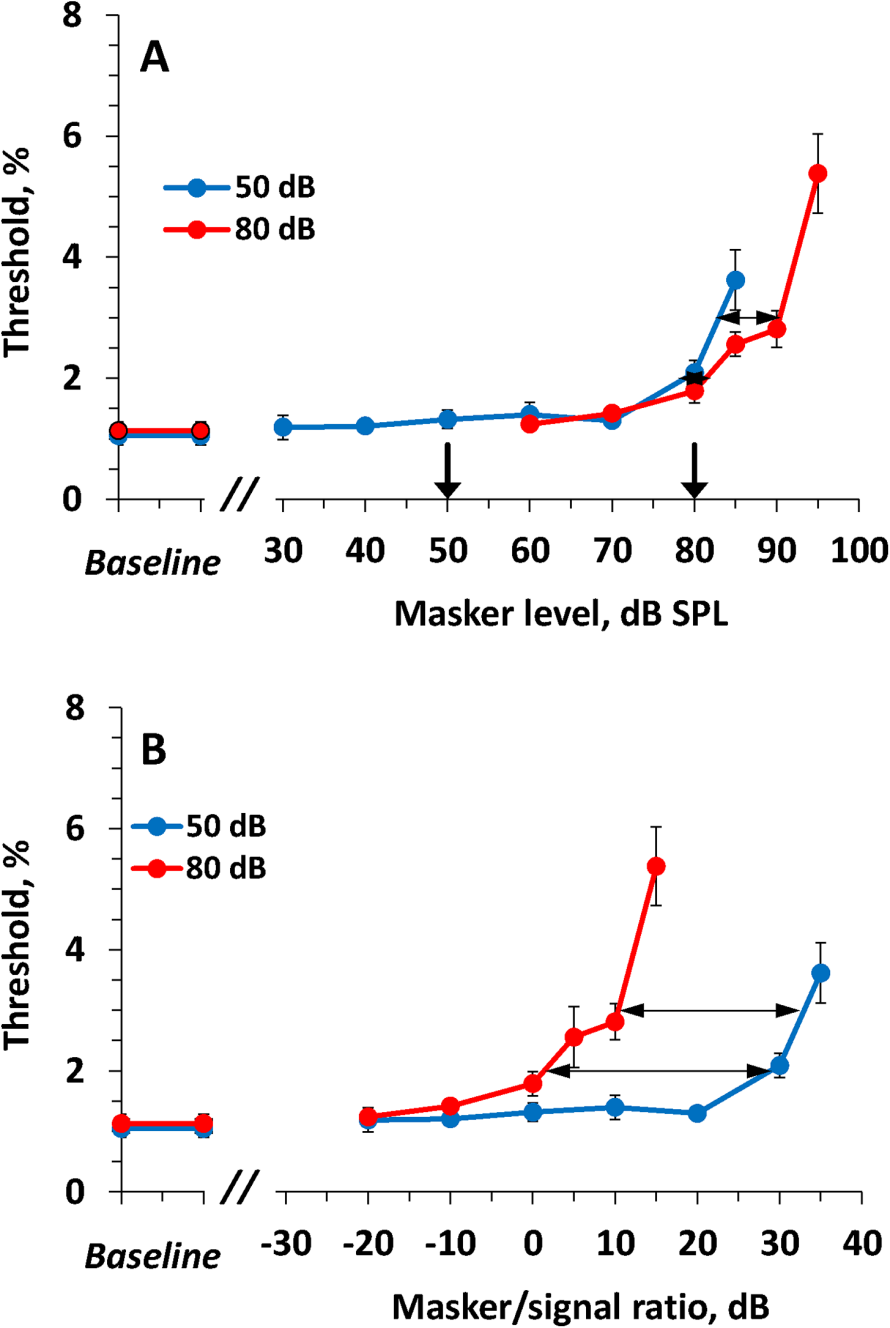
The same as Fig 4 for the low-frequency masker. Variation of masker/signal ratio keeping masker SPL constant (by change of signal SPL by 30 dB) resulted in minor change of thresholds (A) whereas variation of masker SPL keeping masker/signal ratio constant (by joint variation of masker and signal SPLs) resulted in gross change of thresholds (B).

Effects of the low-frequency maskers were investigated for the same two levels of the test signal, 50 and 80 dB SPL. The masker levels were varied from 30 to 100 dB SPL. The results are presented in Fig 5. Similar to the on-frequency maskers, at certain levels, the low-frequency masker resulted in an increase of ripple shift threshold. However, the dependence on both signal and masker levels was different from that for the on-frequency maskers. For both signal levels (50 and 80 dB SPL), differences from the baseline threshold were first observed at a masker level of 80 dB SPL (Fig 5A). The increase (mean ± SE) at the 50 dB SPL signal was 1.04 ± 0.20% (*p* < 0.001), and the increase at the 80 dB SPL signal was 0.66 ± 0.08% (*p* < 0.001). The thresholds increased more with further increase in masker level. For the 50-dB SPL signal, the threshold rose to 3.6% at a masker level of 85 dB SPL, and no ripple shift up to the available limit of 10% was detectable at a masker level of 90 dB SPL; for the 80-dB SPL signal, the threshold rose to 5.4% at a masker level of 95 dB SPL and no ripple shift was detectable at a masker level of 100 dB SPL.

The differences between the functions (for 50-dB and for 80-dB signals) were 4.0 and 8.0 dB for threshold values of 2 and 3%, respectively, with a mean of 6.0 dB.

The same data presented as threshold versus masker/signal ratio (Fig 5B), showed an obvious dissimilarity between the functions for the 50 and 80 dB signal levels. The shift of the two functions relative to one another was 27.5 and 22.0 dB for the threshold values of 2 and 3%, respectively, with a mean of 24.8 dB.

### Effects of high-frequency maskers

**Fig 6:**
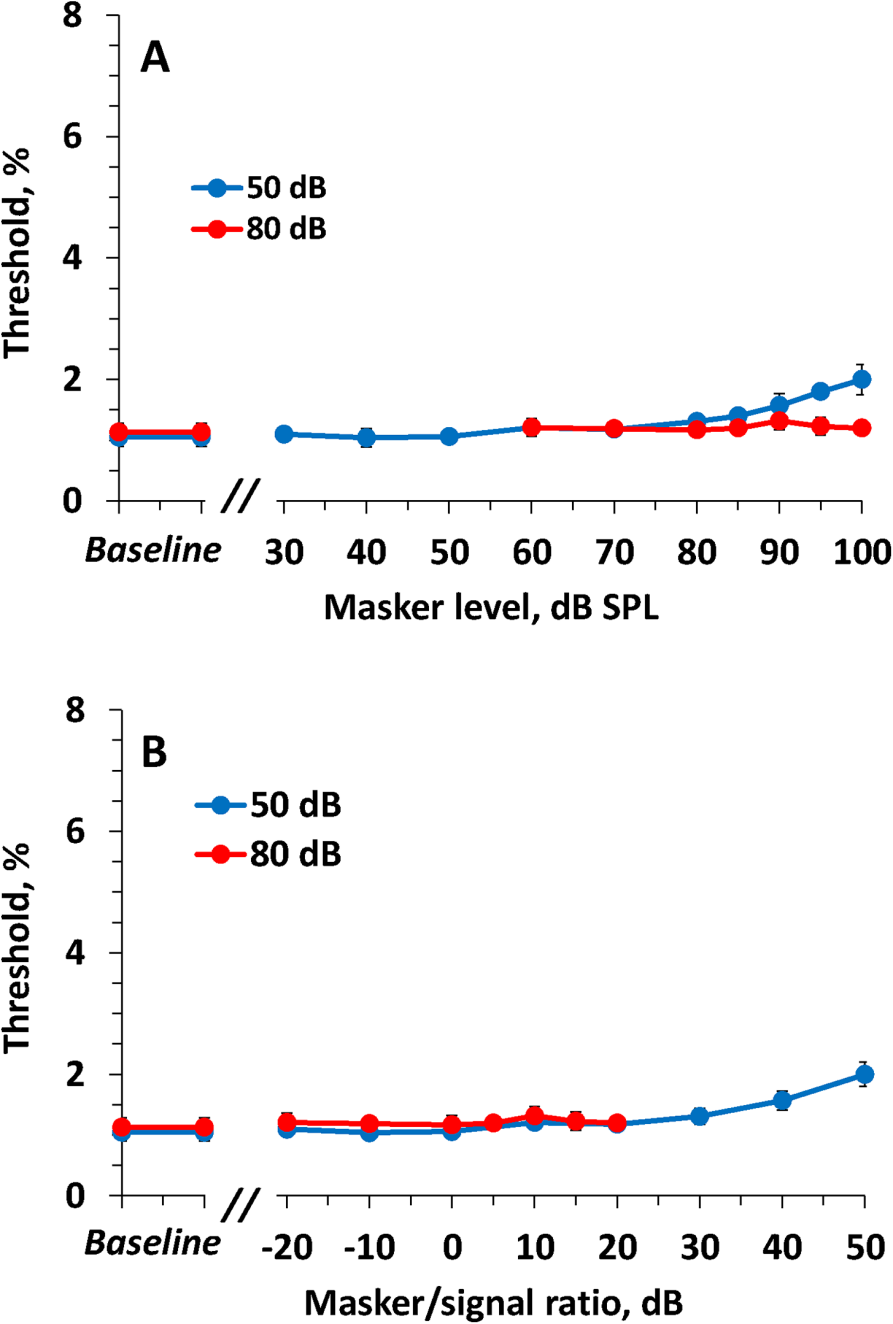
The same as Fig 4 for the high-frequency masker. The effect of masker was negligible except the highest (>30 dB) masker/signal ratios.

Within a wide range of masker levels and masker/signal ratios, the high-frequency maskers hardly affected the ripple shift threshold. Within a masker/signal ratio range from –20 to 30 dB, for both 50-dB and 80-dB test signals, the masked threshold did not significantly differ from the baseline level (from *p* = 0.09 to *p* = 0.74 for the 50-dB signal; from *p* = 0.35 to *p* = 0.63 for the 80-dB signal). At the highest masker/signal ratios (40 to 50 dB), a small threshold increase of up to 2% of the center frequency (*p* = 0.002 for the 50-dB signal and 90-dB masker; *p* < 0.001 for the 50-dB signal and 100-dB masker) was observed (Fig 6).

## Discussion

### Data summary

The data presented above showed that not only on-frequency but also low-frequency maskers deteriorate discrimination of spectrum modifications. Effects of both on- and low-frequency maskers were earlier described for discrimination of pure tones. Experiments with notch-noise maskers revealed masking within a wider range in frequency discrimination than in signal detection [7] with more effective masking by the low-frequency part of the notch noise, so it was suggested that the low‐frequency tail of the excitation pattern is more useful for frequency discrimination than is the high‐frequency tail [8]. Efficiency of low-frequency maskers was also demonstrated by a finding that frequency discrimination is poorer in low‐pass noise than in white noise that combines both low-frequency and on-frequency bands [9]. High-frequency maskers were little effective [10].

These general rules were also characteristic of the masking effects on ripple-shift discrimination as described above. Apart from that, the following features of the masking effects on ripple-shift discrimination may be emphasized:

- The effect of *on-frequency* maskers was markedly dependent on the masker/signal ratio, with little dependence on the masker SPL. When both the masker and signal levels were changed by 30 dB (from 50 to 80 dB SPL), maintaining a constant masker/signal ratio, the change in the masking effect was only a few dB (see Fig 4).
- Unlike on-frequency maskers, the effect of *low-frequency* maskers was markedly dependent on the masker SPL, with little dependence on the masker/signal ratio. When the signal level was changed 30 dB (from 50 to 80 dB SPL), maintaining a constant masker level, the change in the masking effect was only a few dB (see Fig 5).
- The *high-frequency* masker produced minor effects within a wide range of both masker levels and masker/signal ratios (see Fig 6).

### Data modeling

Modeling of the masking effect described above must reproduce the principal differences between the dependence of the masking on level for on- and low-frequency maskers and minimal effects of high-frequency maskers. Popular models of frequency discrimination imply two mechanisms: the excitation pattern (place) processing and the temporal processing [5, 19]. The temporal processing successfully explained the frequency discrimination of pure tones with frequencies below 4–5 kHz. Data on differential thresholds for time separation pitch of wide-band rippled noise with equally spaced ripples were also explained by the temporal processing [20]. The model assumed that although rippled noise does not feature a definite temporal structure, and its hidden temporal structure may be extracted from the autocorrelation function, thus making possible the temporal analysis. However, unlike wide-band rippled noise with equally spaced ripples, narrow-band rippled-spectrum signals with frequency-proportional ripples has an autocorrelation function that do not feature the dependence of ripple shift thresholds on ripple spacing, so it could not be exploited for ripple shift discrimination [11].

The excitation-pattern mechanism better explained ripple shift discrimination for bandpass noise with frequency-proportional ripples. This widely adopted model assumes that, within the auditory system, an excitation pattern (excitation-vs.-frequency function) is produced as the input spectrum passes through a bank of bandpass filters. It was assumed that a stimulus change is detectable when the excitation pattern change exceeds a certain criterion anywhere within the excitation pattern. This model was successfully used for explanation of frequency discrimination limens [21]. Therefore, we tested whether this model could account for the experimental data presented above.

**Fig 7:**
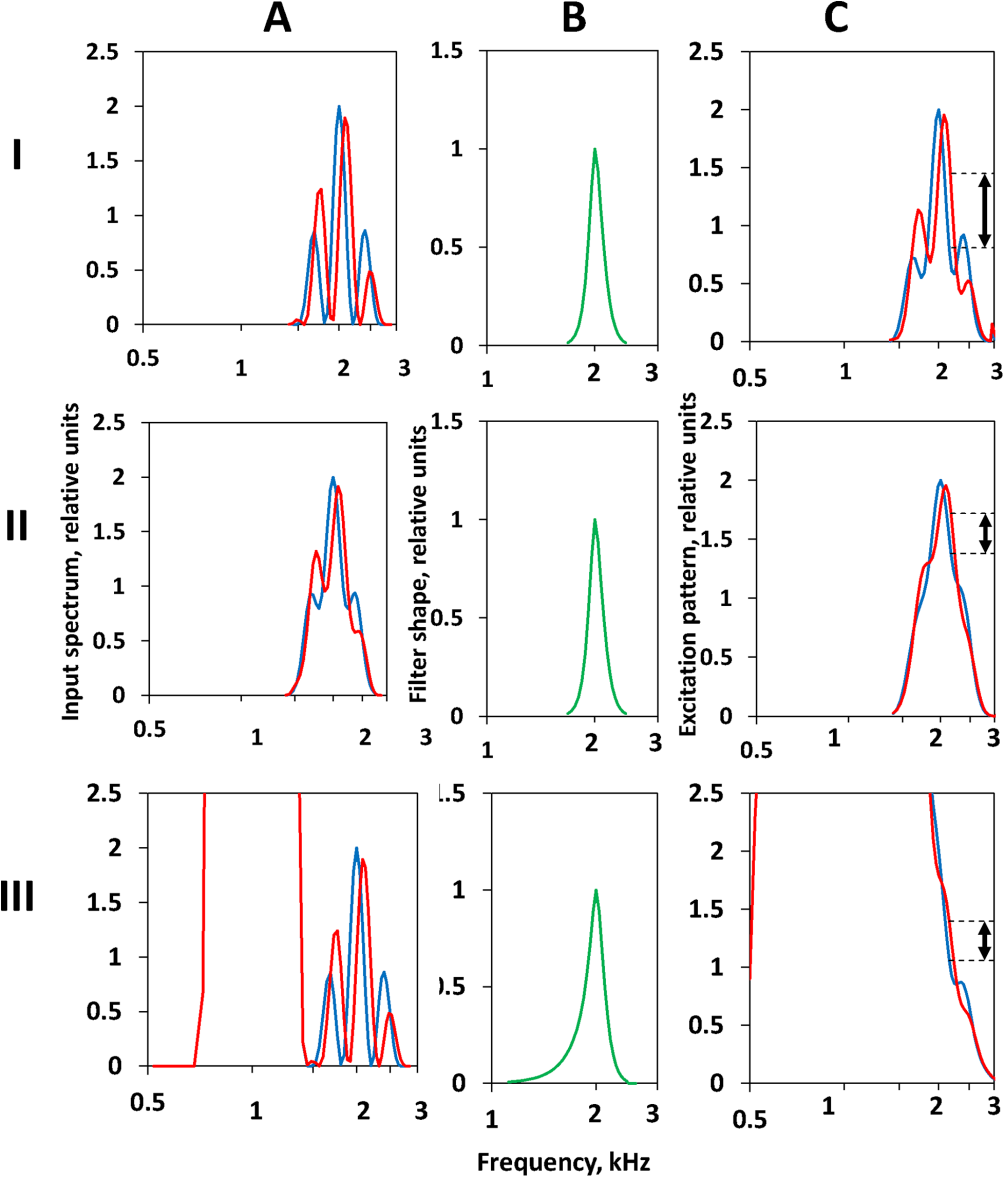
Examples of the derivation of the excitation patterns. (A) Input spectra. **(B)** Filter shape centered at 2 kHz. **(C)** Excitation patterns. **I**. No masker, filter tip gain 30 dB. **II**. Signal plus on-frequency masker, masker/signal ratio 5 dB, filter tip gain 28 dB. **III**. Signal plus low-frequency masker, masker/signal ratio 20 dB, filter tip gain 0 dB. Blue and red lines—input spectra and corresponding excitation patterns with ripples shifted relative one another. Double-headed arrows in (C) mark the detectable shift of the excitation pattern. The on-frequency masker reduced the ripple depth in the signal (A, II) as compared to the non-masked signal (A, I) and the excitation pattern showed a corresponding reduction of the ripple depth (C, II) as compared to the non-masked pattern (C, I). The low-frequency masker did not overlap the signal (A, III), however overlap did occur in the excitation pattern due to transfer through the filter tail (B, III), thus reducing the ripple depth in the excitation pattern (C, III).

We tested a version of this model (Fig 7) wherein the rippled input spectra (A) underwent transfer through a bank of bandpass filters (B) to generate excitation patterns (C). The excitation pattern was obtained by convolution of the input spectrum (A) and the filter shape (B). The filter shape was based on rounded-exponential (roex) functions [22]. According to [15, 16], the filter shape can be represented as the sum of two roex functions: the level-dependent tip (representing the active cochlear mechanism) and the level-independent tail (representing the passive cochlear mechanism). The roex parameters were taken according to the model [16]. Specifically, the roex parameter for the upper half of the tip *p*(*u*) = 28.3, the roex parameter for the lower half of the tip *p*(*l*) = 39.2, the roex parameter for the tail *t* = 13.8, and tip gain *G* varying from a maximum value *G*_max_ to 0 dB as the input level varies from 0 to100 dB SPL. *G* dependence on input level was assumed according to a formula by [16]; that formula reproduces compression for input levels from about 20 to 80 dB. *G*_max_ was adjustable when the model was fitted to the data.

Several examples of the output of this model with the parameters specified above are presented in Fig 7. The excitation patterns (Fig 7C) were modeled using convolution of the input spectrum (Fig 7A) and the filter shape (Fig 7B). It was assumed that the ripple shift could just be detected when shift in the excitation level in any part of the pattern exceeded a certain criterion.

A non-masked excitation pattern is presented in Fig 7(I). For signal levels below 75 dB SPL, the gain *G* was assumed more than 10 dB. At this *G*, the filter equivalent rectangular bandwidth (ERB) is 0.12 of the center frequency (~0.17 oct). At a ripple density of 3.5 rpo, there was a reduction of the ripple depth in the excitation pattern as compared to the input signal. Nonetheless, the ripples were present in the excitation pattern.

The addition of an *on-frequency masker* to the signal reduced the ripple depth (Fig 7A, II). The excitation profile showed a corresponding reduction of the ripple depth (Fig 7C, II). At a reduced ripple depth, a larger ripple shift is necessary to reach the detection criterion. Until the filter ERB is not markedly widened, the reduction of the ripple depth in the excitation pattern and the increase of the threshold depend more on masker/signal ratio than on the masker level.

The spectrum of the *low-frequency masker* did not overlap with that of the test signal (Fig 7A, III). A key assumption to reproduce the experimental data was that the gain *G* depended on the level of both the signal and the low-frequency masker (the upward spreading lateral suppression). The high masker level reduced the gain *G* of the filter tip and increased the weight of the filter tail (Fig 7B, III). The widening of the filter resulted in two effects. (i) Although the masker band did not overlap with the test band in the input signal (Fig 7A, III), overlap within the excitation pattern did occur due to transfer through the filter tails, and this overlap reduced the ripple depth in the excitation pattern (Fig 7C, III). (ii) Filter widening further decreased the ripple depth. As a result, a larger ripple shift was necessary to reach the detection criterion, i.e., the ripple shift threshold increased. Notably, filter widening depends more on masker and signal SPLs than on the masker/signal ratio.

The spectrum of the *high-frequency masker* did not overlap with that of the signal, and unlike the low-frequency maskers, the high-frequency masker was assumed not to decrease the gain *G* at any level. Based on these assumptions, the high-frequency masker did not change the ripple shift threshold compared with the baseline.

Using the procedure illustrated in Fig 7, the effects of maskers on ripple shift thresholds were simulated at various detection criteria and various maximal filter-tip gains *G*_max_. A criterion of 1 dB was previously assumed for modeling of frequency discrimination limens [19, 21] and was consistent with data on profile-depth discrimination thresholds [23–26]. Other authors [27–29] suggested criteria of 2 to 3 dB. Therefore, the model was tested at criteria of 1, 2, and 3 dB. For each of these criteria, the model was tested at *G*_max_ of 20, 40, and 60 dB. A closer approximation to the experimental data was achieved at a discrimination criterion of 1 dB and *G*_max_ of 60 dB.

**Fig 8:**
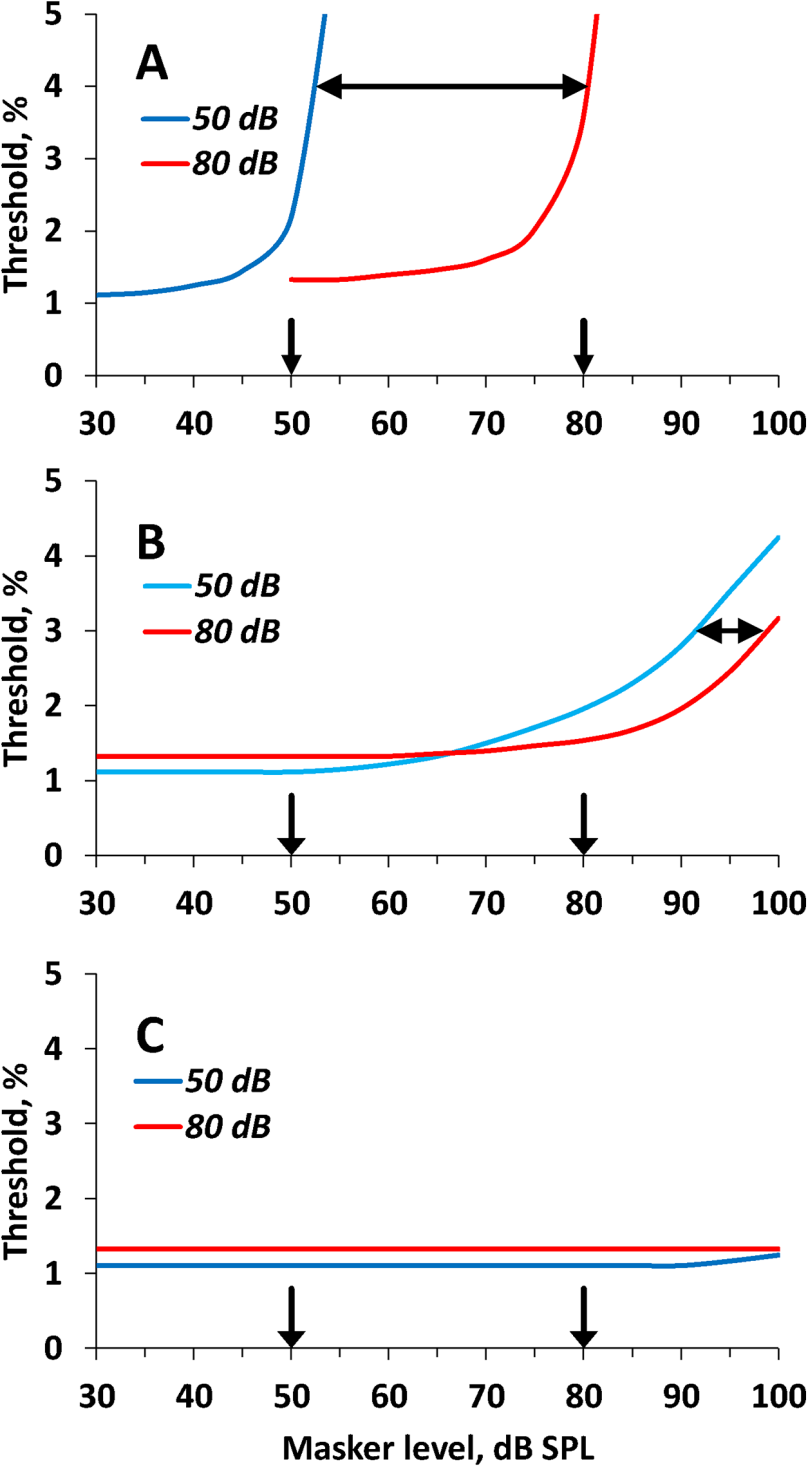
Simulated dependence of threshold on the masker level. (A) On-frequency masker. **(B)** Low-frequency masker. **(C)** High-frequency masker. Plots present threshold-vs-masker level at two signal levels of 50 and 80 dB SPL, as indicated in the legend. Downward pointing arrows depict the levels of the signals (50 and 80 dB). Double-headed arrows in (A) and (B) indicate shifts of the functions for 50-dB and 80-dB signals relative one another. Simulation of the on-frequency masking (A) demonstrated gross threshold dependence on the masker/signal ratio keeping masker level equal (large sift of the plots when the masker/signal ratio was changed by 30 dB) whereas simulation of the low-frequency masking (B) demonstrated minor dependence (minor shift of the plot when the masker/signal ratio was changed by 30 dB). Simulated high-frequency masking (C) demonstrated no or minor dependence on both masker SPL and masker/signal ratio.

This first-approximation model did not precisely simulate the experimental data (Fig 8). In particular, with increasing low-frequency masker level, the threshold increased less steeply than found in experiments (Fig 5). However, the model featured the roles of the masker/signal ratio and masker SPL for the on-frequency and low-frequency maskers.

- The on-frequency masker overlaps the signal thus resulting in the decrease of ripple depth. Therefore, the effect of on-frequency masking primarily depends on the masker/signal ratio. A manifestation of this dependence is the shift of the functions for 50-dB and 80-dB signals relative one another by almost 30 dB (Fig 8A).
- The low-frequency masker does not overlap the signal; therefore, the effect of the low-frequency masker little depends on the masker/signal ratio and primarily depends on the input level because of the filter widening when the level exceeds 70 dB. Therefore, the functions for 50-dB and 80-dB signals are shifted relative one another by only a few dB (Fig 8B).
- The high-frequency masker produced only a minor threshold increase at the highest masker SPL and the highest masker/signal ratios (Fig 8C).

The simulated effects qualitatively resembled the corresponding experimental data (Figs 4A, 5A and 6A). The similarity between the simulated and experimental data suggests similar processes may determine masking effects in the experimental data and explain the differences between the on-frequency, low-frequency, and high-frequency masking effects.

## Conclusion

The influence of maskers on the discrimination of rippled spectra was qualitatively different for on-, low-, and high-frequency maskers. These differences suggest the involvement of different mechanisms. The effect of the on-frequency masker appears primarily due to a decrease of ripple depth produced by the overlapping masker. The effect of the low-frequency masker appears due to lower auditory filter tip gain at high sound levels. The high-frequency masker hardly involves any of these mechanisms. These results might be helpful to predict how spectrum variations of real-world sounds in noisy background are perceived by the auditory system.
